# Evaluating multisite multiprofessional simulation training for a hyperacute stroke service using the Behaviour Change Wheel

**DOI:** 10.1186/s12909-015-0423-1

**Published:** 2015-09-02

**Authors:** AJ Ross, GB Reedy, A. Roots, P. Jaye, J. Birns

**Affiliations:** 1 Glasgow Dental School, University of Glasgow, 378 Sauchiehall Street, Glasgow, G2 3JZ UK; 2 Simulation and Interactive Learning (SaIL) Centre, St Thomas’ Hospital, King’s Health Partners, London, UK; 3 Friends Stroke Unit, Kings College Hospital, Kings Health Partners, London, UK; 4 Department of Ageing & Health, St Thomas’ Hospital, King’s Health Partners, London, UK

**Keywords:** Stroke, Patient simulation, Education, Evaluation

## Abstract

**Background:**

Stroke is a clinical priority requiring early specialist assessment and treatment. A London (UK) stroke strategy was introduced in 2010, with Hyper Acute Stroke Units (HASUs) providing specialist and high dependency care. To support increased numbers of specialist staff, innovative multisite multiprofessional simulation training under a standard protocol-based curriculum took place across London.

This paper reports on an independent evaluation of the HASU training programme. The main aim was to evaluate mechanisms for behaviour change within the training design and delivery, and impact upon learners including potential transferability to the clinical environment.

**Methods:**

The evaluation utilised the Behaviour Change Wheel framework. Procedures included: mapping training via the framework; examination of course material; direct and video-recorded observations of courses; pre-post course survey sheet; and follow up in-depth interviews with candidates and faculty.

**Results:**

Patient management skills and trainee confidence were reportedly increased post-course (post-course median 6 [IQ range 5–6.33]; pre-course median 5 [IQ range 4.67–5.83]; *z* = 6.42, *P* < .001). Thematic analysis showed that facilitated ‘debrief’ was the key agent in supporting both clinical and non-clinical skills. Follow up interviews in practice showed some sustained effects such as enthusiasm for role, and a focus on situational awareness, prioritization and verbalising thoughts. Challenges in standardising a multi-centre course included provision for local context/identity.

**Conclusions:**

Pan-London simulation training under the London Stroke Model had positive outcomes in terms of self-reported skills and motivation. These effects persisted to an extent in practice, where staff could recount applications of learning. The evaluation demonstrated that a multiple centre simulation programme congruent with clinical practice can provide valuable standard training opportunities that support patient care.

**Electronic supplementary material:**

The online version of this article (doi:10.1186/s12909-015-0423-1) contains supplementary material, which is available to authorized users.

## Background

National clinical guidelines in the UK emphasise the need to establish acute stroke as a clinical priority requiring early specialist assessment and treatment [1]. Management on a specialised acute stroke unit from the time of admission results in 19 % more patients being alive and independent at 1 year [2, 3] and ‘clot-busting’ treatment with thrombolysis within 3 h of stroke onset results in 30 % more patients being alive and independent at 3 months [4]. There has thus been increasing recognition of the importance of timely medical attention in acute stroke management [5–7] to facilitate early diagnosis and determination of the aetiology of the stroke (ischaemic or haemorrhagic) in addition to planning treatment strategies aimed at reducing the brain damage caused by the stroke, and preventing complications.

### The London stroke strategy

In 2008, a London-specific stroke strategy was published that made a number of recommendations, including implementation of a new model of acute care incorporating eight hyper-acute stroke units (HASUs) that would deliver care in the first 72 h for all suspected stroke patients [8]. The stroke care model was co-created through a series of events with key stakeholders, clinical experts, patients and carers as well as representatives from carer groups. Subsequent to this wide engagement, the new model was introduced in 2010 with each HASU providing: immediate response; specialist assessment on arrival; brain imaging and thrombolysis (if appropriate) within 30 min; high dependency care and stabilisation. Once stable, the patient is transferred to a stroke unit for rehabilitation and discharge to community care.

The centralised model shows early improvement in patient outcomes [9, 10]. To support its effectiveness, there was an identified training need for the increased numbers of specialist medical and nursing staff recruited to the HASUs.

### The HASU simulation training programme

Following a pilot course, [11] four independent simulation centres provided innovative, multisite training using a standardized protocol-based curriculum based on the London Cardiac and Stroke Network Model [12] and curriculum-mapped against the DoH’s Stroke-specific education framework [13]. The training was designed to provide an immersive, dynamic environment in which learners could practice general and stroke-specific skills without risk to patients [14]. Simulation training is established in healthcare as a valid teaching modality for students, trainees and multiprofessional groups [15]. However multiple-site programmes are rare, as is longitudinal follow-up of candidates [16, 17].

### Aims and objectives

This paper reports on an independent evaluation of the HASU programme. Primary aims were to evaluate design and content, delivery, impact upon learners and transferability to the clinical environment, including making recommendations for faculty development and course improvement.

The main evaluation questions were:

What were the reported behavioural outcomes from the course?

What evidence is there for sustained effect over time?

What recommendations can be made with respect to delivery/evaluation of similar courses?

## Methods

### Conceptualising the training intervention

Michie et al. [18] outline a model, the Behaviour Change Wheel (BCW), for designing and evaluating effective interventions. The ‘wheel’ involves determinations about target behaviours (hub), identification of intervention functions (inner ring) and consideration of policy context (outer ring). Specific behaviour change techniques (BCTs; [19]) are conceptualised as the ‘active ingredients’ by which an intervention achieves its aims. Intervention functions (e.g. training, education) are understood both in relation to the behaviours they target and the policy contexts (e.g. guidelines, regulatory aspects) within which they take place.

In the present study, this model allowed for: a) making the service-provision context explicit b) conceptualising target behaviours; c) studying the behaviour change techniques applied; and d) describing the modes of delivery and findings of the evaluation. Table 1 shows a model of the intervention using the framework. Each simulated exercise and ‘debrief’ was rich in facilitated Behaviour Change Techniques (BCTs); these main agents of change are illustrated together with indications of evaluation metrics.

**Table 1:** Model of the intervention using the Behaviour Change Wheel; specifying policy, intervention and behavioural aspects

BCW model policy level				
Category	Service provision	Fiscal	Guidelines	
Detail	Centralise hyperacute (HASU) care into 8 units situated to provide easy access to the whole population (no more than 30 min by ambulance)	Additional £21 m per year for acute stroke care but only paid under a new tariff if hospitals delivering the required quality	Pan-London Hyper Acute Stroke Nursing Competencies	
BCW model intervention level				
Category	Training; education			
Detail	Simulation training using a standardized protocol-based curriculum based on the London Cardiac and Stroke Network Model			
BCW model behavioural level				
Category	Motivation	Psychological capability	Physical capability	Opportunity
Detail	Forming good habits; increased knowledge and understanding; awareness of role	Cognitive and behavioural (‘non-technical’) skills: communication, management, teamwork	Clinical skills: history taking, assessment, treatment	Resources e.g. calling for help; use of all team members
Main behaviour change techniques (BCTS) in simulation training				
*Repetition and substitution*: habit formation (e.g. taking ‘time-out’ to verbalise situations); practice				
*Goals and planning*: problem solving/coping planning in emergencies				
*Antecedents*: restructuring social environment (e.g. breaking down hierarchies to encourage all voices)				
*Associations*: prompts/cues (e.g. use of critical decision aids)				
*Comparison of behaviour*: modeling; peer review				
*Comparison of outcomes*: pros and cons of different approaches				
*Regulation:* Regulation of negative emotions				
Main outcomes				
Reported knowledge; reported motivational and behavioural outcomes (staff survey and interview data); reported improvement in management and prevention of complications				
Observed data on content, design/learning objectives and delivery				

### Evaluation procedures

Table 2 shows specific procedures undertaken as they relate to various components of the conceptual evaluation model.

**Table 2:** Evaluation procedures mapped to the components of the theoretical framework

Evaluation framework component	Procedures
Policy context	Review of London Stroke Model; Pan-London guidance for stroke protocols; stroke education framework; HASU nurse competencies
Intervention level: training design, content and delivery	Examination of course materials including scenario outlines, learning objectives, presentations, pre-course material
	Direct observation of *n* = 4 HASU course days
	Video and audio playback of *n* = 4 course days
	In depth face-to-face interviews with faculty (*n* = 6)
Behavioural level: behaviours and change techniques	Direct observation of *n* = 4 HASU course days;
	Video and audio playback of *n* = 4 course days;
	In depth telephone interviews with course participants (*n* = 23: 12 doctors; 11 nurses; varying time since course [1–9 months])
	Administered participant surveys before and after the course (*n* = 152)
Outcomes: behaviours and reflections	In depth face-to-face interviews with faculty (*n* = 6)
	In depth telephone interviews with course participants (*n* = 23)
	Administered participant surveys before and after the course (*n* = 152)

Table 2 shows a mixed methods design including before and after survey sheet for trainees (see Additional file 1) and follow-up interviews with staff and faculty. Interviewees were randomly contacted from an attendee list, stratified for basic/advanced course, profession (doctor/ nurse) and time passed since attendance at the course (<3 months; >6 months). Interviewed faculty were chosen purposively, forming a criterion-based sample [20] able to reflect on the design and delivery of the course and its outcomes.

All participants gave prior written informed consent to be contacted for follow-up interview and for survey data to be aggregated for research purposes in accordance with the terms of the Data Protection Act 1998. Ethical approval was given by the Hospital Research Ethics Committee (South London REC 3; approval ref 09/28), under the terms of the UK NHS Research Ethics Service.

All interviews were recorded with permission using a digital voice recorder. The interviews were then transcribed verbatim for data analysis.

### Survey tool

All candidates were given a pre- and post-course questionnaire using 7-point Likert scaled items, adapted from a standard satisfaction measure [21], and some open ended questions. Three scaled items on communication skills, leadership skills and confidence in managing emergency situations were asked identically both before and after the course. Post course perceptions were also gathered on aspects such as course enjoyment and the most valuable learning outcomes.

### Interview tool

Candidate interviews were conducted by telephone and were progressively cued to move from general perceptions to an exploration of specific topics of interest: post-course perceptions; reflections on how the learning objectives were met; what information had been retained; and outcomes in terms of knowledge, skills, personal development/motivation and specific descriptions of patient care episodes.

Faculty interviews were conducted face-to-face according to a semi-structured, topical interview protocol that focused on behavioural needs, training design, delivery and modes of facilitation in the simulated learning environment. The mean interview length was 21 min (candidates) and 29 min (faculty), with a range 16 to 38 min.

### Observation

Observational data were gathered to support the investigators in achieving a complete sense of the scope, scale, and overall experience of the course. Observational data were gathered in three ways:

1. Principal investigators [AR, GR] attended two basic and two advanced courses at multiple training centres and observed all activities.
2. Security permission was established to access audio/video data at one of the centres for the purpose of detailed post-hoc analysis. Data were held on a secure stand-alone drive to protect confidentiality.
3. Secure audio files from a second participating centre were accessed to allow for detailed post-hoc analysis of 6x simulation ‘debriefs’.

### Analysis

Analysis of pre- and post-course survey data took place using appropriate analysis of variance techniques in IBM SPSS v22.0.

Simulation scenarios and debriefs were observed and analysed using SMOTS (Scotia Medical Observation and Training System). Qualitative data from direct observations and interviews and were analysed thematically using HyperRESEARCH 3.5.2 data analysis software. Coding frames were developed from learning objectives and iterated inductively as data were gathered, with discussion of routine and exceptional responses to ensure reliability of cross-coding.

## Results

### Simulation training procedures

The collaboratively-developed multiprofessional programme operated as a ‘basic’ and ‘advanced’ course based on simulated scenarios using a manikin (with computer-controlled vital signs that allowed changes in patient characteristics to be simulated) and/or standardized patient actors. Attendees directly participated in at least one scenario and watched others via a live video-feed. Each simulated scenario lasted up to 15 min and was followed by a group debriefing session lasting approximately 40 min which followed the SaIL debrief diamond model [22] of description, analysis and application to practice [23].

Table 3 shows clinical scenarios employed and specific learning objectives for the basic and advanced courses.

**Table 3:** Curriculum-mapped scenarios and learning objectives

Scenario	Narrative	Course	Objectives
Untreated hypertension	45 year old man admitted to HASU with dysphasia and seizures. CT showed Intracerebral Haemorrhage. The patient has become increasingly restless and the staff over night had difficulty controlling his blood pressure. He is to be rescanned.	Basic	Initial management of hypertensive patient; recognition of acute deterioration; call for help early and appropriately with appropriate tools; equipment required for transfer; appropriate treatment; awareness of complications
Post-stroke seizure	Patient admitted 2 days ago with a haemorrhagic stroke. While nurse is taking a telephone handover about another patient, she is called by a healthcare assistant who has noticed that the patient appears to be twitching	Basic	Recognition of acute deterioration; initial management of seizure; maintains patent airway and administers high flow oxygen; call for help early and appropriately; identifies causes and treatment of a seizure
Hyperacute stroke	73 year old man admitted to HASU at 18:00 last night with fully resolved TIA. Noted by student nurse that patient has new facial weakness. Band 6, Registrar and Consultant available by phone if required.	Basic	Recognition, assessment and management of acute neurological deterioration; call for help early and appropriately with appropriate tools; understanding the importance of urgent escalation
Intracerebral haemorrhage post-thrombolysis	Patient admitted with expressive dysphasia and right sided weakness, National Institutes of Health Stroke Scale (NIHSS) 14. CT scan normal. Thrombolysed (total 76 mg) with good effect. NIHSS at 2 h = 0. The patient appears to have become more confused. Glasgow Coma Score (GCS) deteriorates because of intracranial haemorrhage and oedema	Both	Common presenting symptoms and signs including: nausea, vomiting, headache, altered conscious level, altered pupil reaction, focal deficits of vision, speech, power, sensation; recognition of acute deterioration; understanding of the importance of urgent escalation; appropriate treatment and management of blood pressure
Post-thrombolysis anaphylaxis	Woman admitted to A&E with slurred speech and left sided weakness. Was thrombolysed and transferred to the ward. The band 6 nurse has commenced the altepase infusion and handed the patient over to the ward staff. The patient begins to develop an allergic reaction to the altepase	Both	Calls for help early; administers oxygen and uses bag and mask ventilation safely; monitors; identifies and tries to correct circulatory failure appropriately; identifies potential causes; interprets abnormal vital signs correctly in context; anticipates and prevents deterioration in vital signs
Consent for thrombolysis or breaking bad news using patient actor	45 year old man admitted to A&E FAST positive. For randomisation to new thrombolysis trial, team to gain consent from the patient.	Advanced	Sympathetic, patient-centred approach; discussion of treatment options, complications and side-effects; awareness of consent procedures; assessment of mental capacity; sharing information with patient; breaking bad news
TIA/Stroke examination using patient actor	Received a call from cardiac cath labs at 10:05; patient noted to have new onset of left sided weakness post angiogram at 10:00. Transferred to recovery.	Advanced	Uses NIHSS competently
Thrombolysis for acute ischaemic stroke; patient arriving through A&E	45 year old man admitted to A&E FAST positive. For NIHSS assessment as potential thrombolysis candidate.	Advanced	Assessment of acute focal neurological deficits; use of NIHSS; importance of rapid clinical and radiological assessment; appropriate use of stroke pathway/protocol; appropriate treatment and management of blood pressure/glucose; consent for thrombolysis

Although specific clinical competencies were included, the main learning objectives were more general psychological and physical capabilities (see conceptual model in Table 1): knowledge and understanding (e.g. of stroke signs, symptoms and ‘mimics’, and timeframes for treatments); patient management (e.g. communication skills, team working skills, acting on risk assessment results), and motivational aspects (empowering/enabling staff to increase their confidence in their own professional capabilities).

### Candidates

Seventy-seven candidates attended the Basic HASU course. These were 38 doctors (1–7 years post-qualification) and 39 Registered Nurses at various career stages. Seventy-five candidates with a similar range of seniority attended the Advanced HASU course: 32 doctors and 41 nurses (two missing). All candidates filled in surveys (*n* = 152) but most items have a small amount of missing data.

### Candidate experiences

Overall, candidates enjoyed the course and felt it was relevant to their clinical practice (both items median rating 7/7; IQ range 6–7). Enjoyment and relevance were closely related (Spearman’s rho = .712**; *p* < .001).

Doctors were more likely to rate the course as enjoyable (*z* = 1; *n* = 137; NS), and find it relevant to practice (*z* = 1.2; *n* = 129; NS), than nurses, but a Mann Whitney test for independent groups shows these differences were not significant.

However, this multiprofessional interaction with the scenarios also tended to arise in debrief and in interview. Observations showed nurses having some difficulty in following their usual protocol for assessing patients when the manikin cannot move limbs, does not have a grip response etc. Doctors tended to interact more by talking/taking history (the manikin has voice functionality), by directing treatment, and by reference to notes. This holistic assessment seems more amenable to modelling via simulated practice via manikin than the more direct ‘caring’ provided by nurses (see Discussion). All participants recognised that the manikin gives limited biofeedback cues in providing the context for training realistic stroke care: *because stroke is so dependent on clinical sign things […] is there facial weakness, is this arm moving or not […] it just makes it a little bit false […] I think the thing with an actor is you can replicate stuff a lot […]* (doctor 14); *If you look at somebody, you eyeball them, you can see the difference, you can’t do that with a dummy* (doctor 16); *it’s very hard to look at the symptoms in the manikin […] which is not really manifesting the right things* (nurse 11).

### Capability, motivation and opportunity

Reported competency outcomes were assessed via the survey on three seven-point scale items given before and after the courses: *How good are your clinical communication skills*?; *How good are you leadership skills*?; *How confident do you feel managing emergency situations*? (reliability analysis: Cronbach’s alpha = .897).

**Fig. 1:**
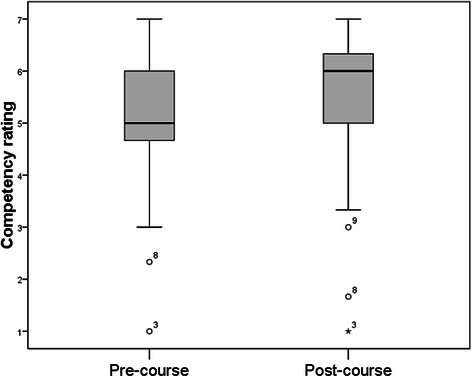
Competency ratings before and after the course (*n* = 141)

Figure 1 shows a comparison of composite scores for these three ‘before and after the course’ items.

Figure 1 shows that these competencies were rated higher after the course.

(Wilcoxon signed ranks test; post-course median 6, IQ range 5–6.33; pre-course median 5, IQ range 4.67–5.83; *z* = 6.42, *P* < .001). Median scores are indicated by the thick line, the box shows the interquartile range (quartiles 2–3), and the ‘whiskers’ show the last scores before outliers (within 1.5 IQR of the lower or upper quartiles).

There were no significant differences (interactions) for course (basic or advanced) or by profession (doctor or nurse) on any of these reported improvements. Overall there was also a slight increase post-course in the perceived usefulness of particular ‘early warning’ scoring systems employed during the training (for those who *n* = 111, *z* = 6.42, *P* < .05).

Qualitative data from survey, interview, and video observations were examined to explore this reported learning further, and to look specifically at the behavioural change techniques employed. Behaviours identified can be grouped thematically into five specific areas: *verbalising thoughts; calling for help; teamwork; assertiveness; and situational awareness.*

Table 4 shows these five main behavioural themes and change techniques employed, with examples of self-reported outcomes synthesised from the survey, interview, and video/audio file observation of the training episodes (all quotations are verbatim).

**Table 4:** Behaviours and change techniques identified, with examples of self-reported learning

Behavioural themes	BCTs employed (from Michie et al. [18])	Detail of delivery	Quotations: Interview [I]; Survey [S]; Audio/Video observation [AV]
Verbalising/sharing the mental model	Habit formation/self and peer monitoring/verbal persuasion/taking time out/feedback on behaviour	Peer-review of videos/identification of critical points/discussion of risk and the importance of speaking out loud and taking timeout for an overview	Thinking aloud sounds like a good technique (Doctor S);
			Sometimes when you’re trying to get to the bottom of problem, somebody might say something and, you know, it triggers a thought process (Doctor I);
			Talking out loud so it is obvious what I am doing, the plan, and what is needed (Nurse S);
			I stepped away from the patient a little bit and said “right, what are we going to do next” (Doctor AV)
Good communication	Peer monitoring/social consequences/modeling/feedback on behaviour	Videos and presented materials/discussions of two-way communication/importance of documenting communication	To ensure communication in events is loud and clear between the team (Nurse S);
			One of the learning points is just how difficult it is for telephone conversations to provide useful results to both sides (Doctor AV)
			The communication skill for a rapid interaction has to be borderline pedantic (Doctor I);
			Communications skills is really, really important, and someone has to listen and someone has to lead (Nurse I)
Managing and planning	Modeling/peer review/problem solving/coping planning/feedback on behaviour	Timelines of scenarios/identification of exemplars/elicitation of strategies employed in practice	The A&E and the stroke team can actually work as a team to actually achieve that door to thrombolysis time of 10 min… To change the practice I would probably get the A&E consultants and the A&E matron to actually be involved in this management of stroke so that the delivery of care can be given within the target time (Doctor I);
			I’ve got this new mindset of going in, that I want to go in and it’s about being mentally prepared for any situation (Nurse I);
			it’s quite difficult to (plan ahead) because you have your own patient to look after, and at the same time co-ordinate the ward (Nurse AV);
			You need to know when to call for help, and when you are at the limit of what you can do on your own (Doctor AV)
Breaking down institutional barriers	Restructuring social environment/self-affirmation/reframing/identity/emotional consequences/pros and cons/social support/feedback on behaviour	Multiprofessional interactions/video review and discussion of leadership and followership/benefits and difficulties of speaking up to senior colleagues	Being a little more assertive, a little more proactive if not happy (Nurse S);
			Human Factors- very interesting dynamic … nobody wants to be the first to say… because, what if you’re wrong? (Doctor AV)
			Someone might not be more senior in the old fashioned hierarchical structure but at that moment in time is more ‘senior’ to you (Doctor AV)
Use of decision aids/tools	Prompts/cues/feedback on behaviour	Discussion/presentation of materials: checklists and clinical decision aids	Luckily … they’ve got protocols plastered up everywhere and when you do say… ‘get the protocol for that’ it appears (Nurse AV);
			[I] made myself a little bit of space and went back to my ABC (Doctor AV)
Situational awareness	Restructuring physical environment/comparative imagining/conserving mental resources/feedback on behaviour	Video playback/discussion/focus on environmental cues and selective attention	Check where the anaphylaxis box is (Doctor S);
			People can get focused on one thing […], focused on one issue and miss out other important things […] (Nurse I);

The main general mechanism for addressing behaviour comes via the post-exercise ‘debrief’, facilitated by senior faculty using the events that have just been observed as a platform. Post-hoc descriptions of scenario timelines and/or use of video playback are used to facilitate peer-group discussions of strategies employed, alternative approaches, personal experiences, emotional aspects and action points to take away. One nurse stressed the importance of the video playback and reflective ‘debrief’ mechanisms:

*We had the scenario played back [and could see] there was a period where we all in the midst of trying to get things done and there was no focus on the patient themselves. So it was quite interesting seeing that because obviously when you’re doing something you just focus on whatever you’re doing, […] you can’t see it from the outside until it’s actually played back. I think playback is quite useful.* (Nurse 13)

Anxiety was felt variably by candidates, but for most was formative, in that it allowed for practice in what would be a stressful situation in real life: *it’s good to be put in that situation I guess, because when you’re in a real life scenario that’s what you need to be able to do sometimes* (Doctor 17). This is a behavioural change agent in itself, via the regulation of negative emotions.

### Delivery and context

The course was designed and marketed as a training event with educational content, however it can also be seen that there is a persuasive element (many discussions focused on how people felt, what they thought patients would feel) and a modelling element (senior faculty provide an example to aspire to). In part, what people ‘took away’ depended on their prior expectations. People who came looking for detailed clinical knowledge of stroke medicine tended to ask clinical questions, take notes, request protocols etc. and thus formed outcomes in their own ideal, taking away technical/skill-based messages (*“noticing blood pressure changing, […] whether to give Labetalol, when to put on the Alteplase, we were experiencing different ways that we deal with this”*). Others who were experienced stroke practitioners tended to have more non-clinical discussions about teamwork and situational awareness. For example senior nurses said of the course: *“made you think on your feet”; “was more about effective communication […] than actual stroke care”; “it was more about managing situations to me”; “it was just reinforcing to be clear and focused on what you were doing […]”.*

Faculty raised a number of issues that apply to similar courses that seek to work in a standardized way across multiple centres, including the balance between overall standardization/reliability and courses being tailored to fit in with the ethos, facilitation style and corporate identity of each individual centre.

### Application to post-course practice

Follow up interviews were intended to explore whether there was any reported transference of simulation-based knowledge and skills to practice. Good application of learning in practice was reported, with candidates specifically recalling: refocusing on ‘door to needle’ time from presentation to treatment; prioritizing during a thrombolysis call; the pertinence of the stroke course to using stroke scoring systems; confidence in initiating stroke management; and verbalizing or vocalizing thoughts in stressful situations.

Most candidates were enthusiastic about the experience and its general motivational effects (*I think it’s a very good experience […] it stimulates you and gets you to get everyone else enthusiastic,* Nurse 2).

More recent attendees were quite explicit about enhanced capability (technical and psychological skills). As one nurse attending a thrombolysis call reported: *I was just imagining the situation from [simulation], so that made it really quite, it made me feel quite confident to do it, because I knew exactly what I was looking for in terms of […] watching out for the signs of anaphylaxis and then monitoring throughout, just to ensure there was no deterioration. […] Just a general sense of, I’ve done this, I just did this in simulation. I can do it again… I was ready for the situation, do you know what I’m saying? If ever they had had a reaction, I was really clear in my mind of how I would actually react to that* (Nurse 3). A doctor had a similar feeling post-course after having helped in a critical care situation: *Yesterday’s case we had in, in resus- it was pertinent having been on the course, getting the stroke team down quickly and starting the scoring system and whether the patient would be thrombolysis or not* (Doctor 9).

### Sustained effects

Qualitative follow-up data show that the course was a driver for ongoing reflective practice, even when, at around 6 months post-course, the ability to recall specific messages or learning ‘on the day’ was seen to degrade. In one instance, a nurse explained how she and her colleagues had instituted changes to their practice for stroke patients after first one, and then several, of them had attended the course and subsequently had time to compare their experiences: *It’s assessing, assessing how we can get our time down, but still getting everything done […] trying to get the ‘door to needle’ time down, but also not missing anything, because you still also need to get your patient’s history […]* (nurse 2).

Overall, confidence was reportedly increased in the months following the course and reflected the various behavioural themes in Table 4 such as verbalising thoughts (*I think, sometimes when you vocalise aloud your thoughts, I think, you know, even if you don’t have the answer you might trigger a thought process from somebody else on the team;* Doctor 7), managing situations (*because of the course I felt more confident in initiating management that maybe other house officers wouldn’t have been able to do;* Doctor 1); situational awareness (*it’s being aware of my surroundings, knowing who I’ve got, just making sure… you’ve got to be specific and use the people that you’ve got there and according to the skills that they have*; Nurse 13).

## Discussion

This paper reported on a structured evaluation of a multi-centre simulation training programme for hyper acute stroke medicine.

### Post-simulation effects

Candidates rated content and design highly in mixed response survey sheets. Candidates reported increased confidence after training. This has been consistently reported across a range of clinical scenarios and specialties [24]. Open-ended responses show nurses reported specific learning based on assertiveness, and were receptive to discussions about social barriers to communication in multiprofessional teams. This training encouraged them to be aware of situations where information flow may be restricted, leading to takeaway messages about what has been termed ‘flattened hierarchy’ [25].

We have reported some general positive evaluations both in post-course survey and follow up interviews, and some specific relation of the intervention and its mechanisms of change to improved behaviours in the new service delivery environment.

Following up after time has elapsed in important, because transfer to practice [26] and the sustainability (or decay) of training-acquired skills or knowledge over time [27] has been a relatively neglected area of simulation research [28, 29]. Thus, “[…] some of the challenges that still exist in simulation-based medical education include […] measuring the effect of simulation and the transference of knowledge from the simulated environment to real life” [30].

### Capability, motivation and opportunity

The evaluation was designed to study how the programme actively addressed capability, opportunity and motivation. We have reported that learner expectations vary with respect to whether they are learning skills or acquiring knowledge. Motivational effects (e.g. reported change of ‘mindset’) also emerge during post-course reflection. Despite growing use of simulated modalities and positive evaluations, relatively few simulation studies have used a theoretically driven evaluation within which intended outcomes can be framed. It is important that trainers are clear whether each episode is targeting physical/technical and/or psychological skills, motivation, opportunity (including social) or combinations of all three.

In particular, some candidates (those less experienced in clinical management of stroke) expected knowledge-based outcomes and others (experienced) expected to focus on extended skills. It is important that simulation designers and facilitators anticipate multiprofessional differences in receptivity and that this feeds back into design so that learning objectives are tailored to specific student needs [31]. Learning from participatory simulation must address contextual and systems factors, which in turn give rise to emergent outcomes [32]. There are ongoing discussions in the literature about compatibility of different learning outcomes that tend to be mixed in simulation, based on knowledge or skill acquisition and/or the aim to provide transformative personal experience [33].

Further, we have identified some important behavioural change techniques that recur in simulated performance (and in particular in peer ‘debriefing’). These include forming good habits such as verbalisation and taking ‘time outs’, and social restructuring around professional hierarchies, to encourage speaking out about safety.

However we also identified variance in debriefing styles and approaches, despite standard materials and learning outcomes. Cantrell (2008) reports that different styles *per se* are not problematic [34] as long as, as in this study, debriefing takes place immediately following scenarios while perceptions are still salient. However active engagement of candidates is key [35], and some faculty reports suggested some styles were more didactic in nature. Results also showed that there were also some senior clinical faculty who were not trained specifically in simulation debrief (especially with relation to non-clinical or ‘non-technical skills’) and this has been recognised as important for best practice [36].

Michie et al. [19] conclude that further ‘elucidation of how content, mode, and context of delivery interact in their impact on outcomes is a key research goal for the field of behavioral science’. The physical environment in this mode of training delivery is not ‘the same for everyone’. Observations and interviews showed an interaction whereby fidelity was reportedly more of an issue for nurses. Nurses had more difficulty in following their usual protocol for assessing patients; the manikin cannot move limbs, does not have a grip response etc. Doctors tend to perform in a space less contiguous to the manikin via taking history (the manikin has voice functionality), directing treatment and referring to notes. It may seem self-evident that “fidelity is the degree to which a simulation replicates or approaches reality” [37], but there are various social and psychological dimensions that need to be taken into account and it is not easy in applied courses of this type to assess fully whether thresholds for fidelity are being met [38].

### Strengths and limitations

Results in this paper are supportive of standardised multiprofessional training for stroke medicine and indicate benefits. Reported post-course confidence ratings, for example, are increased and internally reliable (as in previously reported studies [39]) but these are insufficient in themselves as evidence for patient benefit. We have triangulated findings with follow-up interviews on reports about the use of these skills in practice.

The next stage might be to examine, for example using case comparison, patient outcomes in units using standard educational modalities against simulation training. This in effect is a complex intervention and attribution of variance in outcomes to specific training events is difficult, but empirical tests of context-mechanism-outcome configurations have been recommended [40].

## Conclusions

Data show that pan-London simulation training under the London Stroke Model has positive outcomes for staff in terms of their emotional reactions and self-reported behavioural outcomes, both in terms of skills and motivation. These effects persist to a certain extent in practice, where staff can recall training episodes and change engendered. Simulation ‘debriefing’ after live video recorded scenarios offers many possibilities for tailored behaviour change techniques; trainers should be clear about a) target behaviours/learning objectives, and b) specific mechanisms of change. Simulation training was effective in helping achieve HASU-specific learning outcomes and the project demonstrated that a carefully designed simulation programme congruent with clinical practice can provide valuable training opportunities that support patient care.

## Additional file

Additional file 1: **Acute Stroke Simulation Training Questionnaire.** (DOC 157 kb)

## References

[1] Royal College of Physicians Intercollegiate Stroke Working party: National Clinical Guidelines for stroke. 4th Edition 2012. [http://www.rcplondon.ac.uk/sites/default/files/national-clinical-guidelines-for-stroke-fourth-edition.pdf]

[2] Kalra L, Evans A, Perez I, Knapp M, Donaldson N, Swift CG (2000). Alternative strategies for stroke care: a prospective randomised controlled trial. Lancet.

[3] Evans A, Perez I, Harraf F, Melbourn A, Steadman J, Donaldson N (2001). Can differences in management processes explain different outcomes between stroke unit and stroke-team care?. Lancet.

[4] National Institute of Neurological Disorders and Stroke rt-PA Stroke Study Group (1995). Tissue plasminogen activator for acute ischemic stroke. N Engl J Med.

[5] Stone S (2002). Stroke units. Br Med J.

[6] Harbison J, Hossain O, Jenkinson D, Davis J, Louw SJ, Ford GA (2003). Diagnostic accuracy of stroke referrals from primary care, emergency room physicians, and ambulance staff using the face arm speech test. Stroke.

[7] Nor AM, Davis J, Sen B, Shipsey D, Louw SJ, Dyker AG (2005). The Recognition of Stroke in the Emergency Room (ROSIER) scale: development and validation of a stroke recognition instrument. Lancet Neurol.

[8] Healthcare for London. Stroke Strategy for London. [http://www.londonhp.nhs.uk/wp-content/uploads/2011/03/London-Stroke-Strategy.pdf]

[9] Hunter RM, Davie C, Rudd A, Thompson A, Walker H, Thomson N (2013). Impact on Clinical and Cost Outcomes of a Centralized Approach to Acute Stroke Care in London: A Comparative Effectiveness Before and After Model. PLoS ONE.

[10] Morris S, Hunter RM, Ramsay AIG, Boaden R, McKevitt C, Perry C, et al. Impact of centralising acute stroke services in english metropolitan areas on mortality and length of hospital stay: difference-in-differences analysis. Br Med J. 2002;349:4757. doi:10.1136/bmj.g4757. 10.1136/bmj.g4757 PMC4122734 25098169

[11] Roots A, Thomas L, Jaye P, Birns J (2011). Simulation training for hyperacute stroke unit nurses. Br J Nurs.

[12] NHS London Cardiac and Stroke Networks. The London Stroke Model. [http://www.slcsn.nhs.uk/uksf/stroke-forum-lsm1.pdf]

[13] Department of Health. Stroke-specific education framework. [http://www.weds.wales.nhs.uk/sitesplus/documents/1076/Stroke-Specific_E_Framework.pdf]

[14] Reed K, Wood S, Jacobson L, Chang E, Milzman D (2011). Stroke simulation training: is stroke management missing in residency training?. Ann Emerg Med.

[15] del Moral I, Maestre JM (2013). A view on the practical application of simulation in professional education. Trends in Anaesthesia and Critical Care.

[16] Foronda C, Liu S, Bauman EB (2013). Evaluation of simulation in undergraduate nurse education: An integrative review. Clin Sim in Nursing.

[17] Ross AJ, Kodate N, Anderson JE, Thomas L, Jaye P (2012). A content analytic mapping of simulation studies in anaesthesia journals, 2001–2010. Brit J Anaesth.

[18] Michie S, van Stralen MM, West R (2011). The behaviour change wheel: A new method for characterising and designing behaviour change interventions. Implement Sci.

[19] Michie S, Richardson M, Johnston M, Abraham C, Francis J, Hardeman W (2013). The behavior change technique taxonomy (v1) of 93 hierarchically clustered techniques: building an international consensus for the reporting of behavior change interventions. Ann Behav Med.

[20] Patton MQ (1990). Qualitative evaluation and research methods 2nd edition.

[21] Levett-Jones T, McCoy M, Lapkin S, Noble D, Hoffman K, Dempsey J (2011). The development and psychometric testing of the Satisfaction with Simulation Experience Scale. Nurse Educ Today.

[22] Jaye P, Thomas L, Reedy G (2015). “The Diamond”: a structure for simulation debrief. Clin Teach.

[23] Steinwachs B (1992). How to facilitate a debriefing. Simul Games.

[24] Akhu-Zaheya LM, Gharaibeh MK, Alostaz ZM (2013). Effectiveness of simulation on knowledge acquisition, knowledge retention, and self-efficacy of nursing students in Jordan. Clin Sim in Nursing.

[25] Paige JT, Garbee DD, Kozmenko V, Yu Q, Kozmenko L, Yang T (2014). Getting a head start: high-fidelity, simulation-based operating room team training of multiprofessional students. J Am Coll Surg.

[26] Murin S, Stollenwerk NS (2010). Simulation in procedural training. Chest.

[27] Elfrink VL, Kirkpatrick B, Nininger J, Schubert C (2010). Using learning outcomes to inform teaching practices in human patient simulation. Nurs Educ Perspect.

[28] McGaghie WC, Draycott TJ, Dunn WF, Lopez CM, Stefanidis D (2011). Evaluating the impact of simulation on translational patient outcomes. Simul Healthc.

[29] Cant R, Cooper S (2010). Simulation-based learning in nurse education: systematic review. J Adv Nurs.

[30] Birns J, Jaye P, Roots A, Reedy G, Ross AJ (2014). A Pan-London simulation training for hyperacute stroke [abstract]. Stroke.

[31] Kharasch M, Aitchison P, Ochoa P, Aitchison P, Zhao JC, Kharasch M (2011). Growth of a simulation Lab: Engaging the learner is key to success. Dis Mon.

[32] Jordan M, Lanham HJ, Anderson RA, McDaniel RR (2010). Implications of complex adaptive systems theory for interpreting research about health care organizations. J Eval Clin Pract.

[33] Stayt LC (2012). Clinical simulation: A sine qua non of nurse education or a white elephant?. Nurse Educ Today.

[34] Cantrell MA (2008). The importance of debriefing in clinical simulations. Clin Sim in Nursing.

[35] Dreifuerst KT (2009). The essentials of debriefing in simulation learning: a concept analysis. Nurs Educ Perspect.

[36] The INACSL Board of Directors (2011). Standard VI: the debriefing process. Clin Sim in Nursing.

[37] Paige JB, Morin KH (2013). Simulation fidelity and cueing: A systematic review of the literature. Clin Sim in Nursing.

[38] Alessi S. Simulation design for training and assessment. *Aircrew training and assessment.* Edited by O’Neil H, Andrews D. Mahwah, NJ: Lawrence Erlbaum Associates; 2000:197–222.

[39] Liaw SY, Zhou WT, Lau TC, Siau C, Chan SW (2013). An multiprofessional communication training using simulation to enhance safe care for a deteriorating patient. Nurs Educ Today.

[40] Marchal B, Westhorp G, Wong G, Van Belle S, Greenhalgh T, Kegels G (2013). Realist RCTs of complex interventions: An oxymoron. Soc Sci Med.

