# Supplementary material for: Evaluating multisite multiprofessional simulation training for a hyperacute stroke service using the Behaviour Change Wheel
Source: BMC Med Educ. 2015 Sep 2;15:143. doi: 10.1186/s12909-015-0423-1 (PMC4557755; doi:10.1186/s12909-015-0423-1)
Supplement: Additional file 1: — Acute Stroke Simulation Training Questionnaire. (DOC 157 kb) [file 12909_2015_423_MOESM1_ESM.doc]

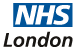

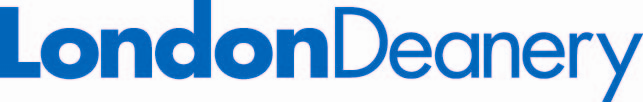


**Acute Stroke Simulation Training Questionnaire**

**Course Date**: _____________ **Venue** _____________ **Your grade/Band**: _____________

**Pre-course items**

*Please circle your preferred answer* for each question.

| What is your level of experience with simulation | 1 2 3 4 5 6 7  Low High |
| --- | --- |
| Patient safety is an issue that needs to be improved from what I have seen during clinical practice | 1 2 3 4 5 6 7  Strongly Strongly  Disagree Agree |

| Have you ever used an early warning score such as PAR or NEWS? | YES NO |
| --- | --- |
| If yes, how useful was it? | 1 2 3 4 5 6 7  Not Very  useful useful |

| **On a scale of 1-7,** how good are your clinical communication skills? | 1 2 3 4 5 6 7 |
| --- | --- |
| **On a scale of 1-7,** how good are you leadership skills? | 1 2 3 4 5 6 7 |
| **On a scale of 1-7,** how confident do you feel managing emergency situations? | 1 2 3 4 5 6 7 |


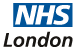

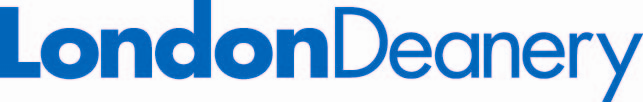


**Post-course items**

Have you been in High Fidelity Simulation Scenarios before? **YES NO** If yes, how many times? _______

How did this experience compare to previous simulation sessions? **Better/ Same/ Worse**

Please rate each component of the course: 1=terrible 7=terrific

| Did you enjoy the course? | 1 2 3 4 5 6 7 |
| --- | --- |
| How relevant was this course to your clinical practice? | 1 2 3 4 5 6 7 |
| Rating for *Introduction to Simulation* | 1 2 3 4 5 6 7 |
| Rating for *Familiarisation with Mr/Mrs Simulator* | 1 2 3 4 5 6 7 |
| Rating for *Simulator scenarios* | 1 2 3 4 5 6 7 |
| Rating for *Catering* | 1 2 3 4 5 6 7 |
| Rating for *Venue* | 1 2 3 4 5 6 7 |
| Rating for *Faculty* | 1 2 3 4 5 6 7 |
| Rating for *Communication session* | 1 2 3 4 5 6 7 |

| Is the early warning score system useful? | 1 2 3 4 5 6 7  Not Very  useful useful |
| --- | --- |

| **On a scale of 1-7,** how good are your clinical communication skills? | 1 2 3 4 5 6 7 |
| --- | --- |
| **On a scale of 1-7,** how good are you leadership skills? | 1 2 3 4 5 6 7 |
| **On a scale of 1-7,** how confident do you feel managing emergency situations? | 1 2 3 4 5 6 7 |

*(Note: all these items open-ended with space to comment):*

Was the course too long or too short?

Was there anything you particularly liked?

Was there anything you particularly didn’t like?

What did we do well?

What could we do differently?

What is one thing you are going to take away from this course?

Additional comments?
